# Expectations regarding transitioning into long-term care, social connectedness, and mental health of older adults

**DOI:** 10.1007/s00127-025-02975-4

**Published:** 2025-08-18

**Authors:** Annalise Lane, Linh Dang, Weidi Qin, Sarah Burgard, Briana Mezuk

**Affiliations:** 1 Center for Social Epidemiology and Population Health, Department of Epidemiology, University of Michigan School of Public Health, Ann Arbor, MI USA; 2 Department of Health Management and Policy, School of Public Health, University of Michigan, Ann Arbor, MI USA; 3 Sandra Rosenbaum School of Social Work, University of Wisconsin– Madison, Madison, WI USA; 4 Department of Sociology, University of Michigan, Ann Arbor, MI USA; 5 Institute for Social Research, University of Michigan, Ann Arbor, MI USA; 6 University of Michigan School of Public Health, 1415 Washington Heights, Ann Arbor, MI 48109 USA

**Keywords:** Mental health, Aging in place, Long-term care, Social characteristics

## Abstract

**Purpose:**

This study examined the association between perceived likelihood of moving into a nursing home and depressive and suicidal outcomes among adults aged 65+, and explored variation in those associations by two aspects of social connectedness: individual social networks and neighborhood social cohesion.

**Methods:**

Data comes from the 2018 Health & Retirement Study (*N* = 7,897). Perceived likelihood of moving into a nursing home in the next five years was assessed using a probability scale (0-100%). Past-year elevated depressive symptoms, major depressive episodes (MDE), and passive suicidal ideation (PSI) were indexed by the Composite International Diagnostic Interview. Social networks (e.g., diversity and frequency of contact with social network, number of close relationships) and neighborhood social cohesion (e.g., living close to good friends, neighborhood social cohesion index) were self-reported. Multivariable logistic regression models were used to examine the association between nursing home expectations and depression outcomes; moderation by social connectedness was examined using interaction terms.

**Results:**

The majority of respondents reported low perceived likelihood of moving into a nursing home (median: 5%, IQR: 0–20%). Higher perceived likelihood was positively associated with depressive symptoms (Odds Ratio [OR]: 1.06, 95% CI: 1.01, 1.11), MDE (OR: 1.08, 95% CI: 1.02, 1.15), and PSI (OR: 1.10, 95% CI: 1.04, 1.17). Having a close friend in the neighborhood heightened the association between expectations and mental health; other measures of social connectedness did not moderate this association.

**Conclusion:**

Older adults anticipating transitioning to long-term care may have unmet emotional support needs, particularly if they are socially-integrated in their neighborhood.

**Supplementary Information:**

The online version contains supplementary material available at 10.1007/s00127-025-02975-4.

## Introduction

More than half of adults aged 65 + will use some form of long-term care (LTC) services [1]. In 2015, one million adults over age 65 (2.6%) lived in nursing homes and two million (4.8%) lived in other LTC residential settings (e.g., assisted living) [2]. The transition into a nursing home or other type of LTC facility can be a prolonged process that is often associated with relocation-related emotional stress [3] and symptoms of anxiety and depression [4]. However, as evidenced by the diversity of long-term care services and supports, several aspects of this transition are modifiable [5]. Therefore, understanding the contextual factors salient to the relationship between LTC transitions and mental health can inform intervention strategies aimed to support mental health of older adults prior to and during these transitions [6].

One important contextual factor salient to the relationship between LTC transitions and mental health is the *expectation* (i.e., anticipation or perceived likelihood) of moving into LTC. These expectations are correlated with established predictors of LTC transitions, including older age and presence of serious health conditions or disabilities [7]. For example, Lindrooth et al. (2000) found that older individuals with more activities of daily living (ADL) limitations reported higher expectations of moving into a nursing home in the next five years [8]. Such expectations strongly predict future transitioning into LTC, even after accounting for physical and cognitive impairments [9]. These findings suggest that expectations regarding moving into LTC may reflect additional information regarding older adults’ personal beliefs and cultural and social norms [10] towards residential care, above and beyond their actual health status, family structure, and care support needs [11–14].

### Expectations regarding LTC transitions and mental health

Theoretical models of anxiety and depression emphasize the roles of uncertainty, cognitive inflexibility, and hopeless views about the future in the etiology of these conditions [15–17]. For example, a cross-sectional study by Assari and Lankarani (2016) found a positive association between depression and hopelessness [18]. Other studies also show that poor mental health is associated with negative outlooks about the future [19] and greater perceived likelihood of future undesirable and/or challenging events [20]. As discussed above, the process of moving into LTC is often associated with a range of negative emotions including suicidal behaviors [3, 21, 22]. For example, Mezuk et al. (2019) found that over 40% of LTC-related suicide deaths referenced the anticipated, in progress, or thwarted transition into or out of residential care [23]. While SAMSHA produces guidance for reducing suicide risk in residential care settings [24], additional research is warranted to examine the relationship between *expectations regarding moving into LTC* and mental health to further clarify intervenable touchpoints for promoting emotional health of older adults during this transition.

### Importance of social connectedness as a determinant of mental health in later life

Social connectedness is characterized by several features that may influence the process of transitioning into LTC, including network diversity (e.g., family, friends, neighbors), frequency of interaction with others, and number of close relationships. Older adults typically have smaller and less diverse social networks compared to the younger adults [24]; however, older adults maintain more frequent contacts with their most intimate social ties (e.g., family members and close friends) [25]. Extensive research shows that social connectedness is associated with mental health [26]; for example, older adults with more diverse social networks reported lower depressive symptoms than those with more restricted networks [27].

Social relationships have an important role concerning care transitions in later life, including efforts to “age in place.” Older adults with complex health needs often rely on their social network for assistance with core activities (e.g., managing finances, running errands), healthcare-related decision-making (e.g., attending or coordinating healthcare appointments), and emotional support (e.g., coping with life adjustments, such as loss of friends or family members) [28]. This suggests that social network characteristics may act as moderators of the relationship between stressful life events, such as transitioning into residential care, and mental health. For example, social relationships buffered against depression among older adults who are at risk for LTC transition such as those experiencing functional limitations [29] and loss of independence [30]. Alternatively, a lack of social connectedness that experienced during or after a nursing home transition may worsen mental health [26], though this theoretical model has not, to our knowledge, been studied exclusively in older adult populations.

Beyond social network characteristics, neighborhood social cohesion (defined as the perceived shared values, sense of belonging, willingness to help, and shared trust among the residents in their neighborhoods [31]) may also buffer against poor mental health during stressful events. For example, Zeng & Wu (2022) demonstrated that neighborhood social cohesion alleviated stress among older adults in the wake of a neighbor’s suicide death [32]. Choi and Matz-Costa (2018) found that neighborhood social cohesion buffered the negative consequences of local crime on mental health among older adults with functional limitations [33]. Other studies showed that neighborhood social cohesion offered protective effects against the mental health impacts of natural disasters among displaced older adults [34, 35]. Together, these studies suggest that social cohesion may also moderate the relationship between expectations regarding LTC transitions and mental health.

### Study objectives

While previous research has documented poor mental health associated with LTC transitions, most of these studies focus on older adults who r*ecently moved into* or are *already living* in such settings, rather than the *expectations* of this transition. In the handful of studies that have examined anticipation of transitioning into LTC, most relied on small, demographically homogeneous samples [21] or death records to indicate whether the decedent anticipated an LTC transition [23]. Additional research is warranted to understand the factors that shape mental health during LTC transitions, particularly among community-residing older adults. Moreover, while there is strong evidence that social connectedness is protective of mental health during major life events, little is known about the potential influence of social connectedness on anticipated residential care transitions in later life.

Therefore, the primary objective of this cross-sectional study was to examine the association between perceived likelihood of moving into a nursing home (hereafter: *nursing home expectations*, or simply “expectations”) among a population-based sample of community-residing adults aged 65 + and three indicators of depressive symptomatology: elevated depressive symptoms, experiencing a major depressive episode (MDE), and reporting passive suicidal ideation (PSI). The second objective was to explore the potential moderating roles of social connectedness, as indicated by social network characteristics and neighborhood social cohesion, on the association between nursing home expectations and mental health. Given that nursing home expectations are strongly predictive of future transition into LTC [9], understanding the relationships between nursing home expectations, social connectedness, and mental health can provide valuable insights into modifiable factors to support older adults during residential care transitions.

## Method

### Data source

Data came from the Health and Retirement Study (HRS), a nationally representative longitudinal survey of US adults over age 50. Since 1992, approximately 20,000 living HRS respondents have been interviewed biennially to assess a range of psychosocial characteristics, health history, and expectations, including expectations of transitioning to a nursing home. The HRS employs a complex, multi-stage design, including oversampling Hispanics, African Americans, and Florida residents, and enrolls additional respondent cohorts every six years to remain representative of the current older adult population. Additional details surrounding the design of the HRS are described elsewhere [36]. The HRS is approved by the Institutional Review Board at the University of Michigan. All respondents provided written informed consent (https://hrs.isr.umich.edu).

The main sample included non-proxy respondents ages 65 + who were interviewed in 2018 (i.e., the most recent wave of the HRS that was not impacted by the COVID-19 pandemic) and had available data on mental health outcomes, expectations of moving to a nursing home in the next five years, and all covariates (*N* = 7,897). The leave-behind subsamples included respondents who had available data on social network (*N* = 3,034) and neighborhood social cohesion characteristics (*N* = 2,577) via completing the 2018 h Leave-Behind Questionnaire (which was administered on a random 50% subsample of the full HRS). Supplementary Fig. 1 details the selection process of the main sample and leave-behind subsamples. Similar demographic and health characteristics were observed for the main sample and leave-behind subsample (Table 1). Compared to the 2018 h respondents excluded due to missing on key variables, included respondents in the main sample were more likely to be older, wealthier, and have at least one physical comorbidity (Supplementary Table 1).

### Exposure: nursing home expectations

Perceived likelihood of moving to a nursing home was assessed only among respondents over age 65 by the question, “What is the percent chance that you will move to a nursing home in the next five years?” Responses were recorded on a probability scale of 0-100%. To generate a more meaningful interpretation of regression coefficients, these responses were rescaled to 0–10% such that a 1-unit increment in the rescaled variable corresponds to a 10-unit increment in the original. In addition, a categorical version of the nursing home expectations variable (operationalized as 0%, 1–10%, 11–40%, 41–60%, 61–100%) was constructed via visual inspection of the histogram of this variable.

### Outcomes: depressive and suicidal symptomatology

This study examined three indicators of depressive symptomatology during the past 12 months: elevated depressive symptoms (yes/no), MDE (yes/no), and PSI (yes/no). These outcomes were assessed by the Composite International Diagnostic Interview-Short Form (CIDI). The CIDI is a fully structured diagnostic interview for past 12-month depressive symptoms based on the Diagnostic and Statistical Manual of Mental Disorders IV (DSM-5) [37]. The CIDI assesses eight symptom domains of depression including sadness, anhedonia/lost interest, sleeping disturbances, appetite changes, fatigue, guilt/worthlessness, concentration problems, and thinking about death. Higher scores on the CIDI indicate more severe depressive symptomatology.

Elevated depressive symptoms were defined as screening into the full CIDI module (i.e., endorsing the feelings of depression or loss of interest at a sufficient frequency and intensity for at least 2 weeks within the past 12 months) [36]. In alignment with DSM-5 diagnostic criteria, MDE was operationalized as endorsing at least five CIDI symptoms [38]. PSI was defined as an affirmative response to whether the respondents “thought a lot about death–either their own, someone else’s, or death in general.” PSI was coded as 0 for respondents who did not answer affirmatively and those who did not meet criteria for elevated depressive symptoms (i.e., screened out of the CIDI and therefore were not asked this question).

### Moderators: social connectedness

***Social Network Characteristics.*** Social network characteristics were assessed by self-report in the leave-behind survey and operationalized as (1) diversity, (2) frequency of contact, and (3) number of close relationships. Diversity measured the number of relationship types the respondents had, including relationships with spouses/partners, living children, other immediate family members, and/or friends (range: 0–4). Frequency of contact, either in-person or by phone, was assessed separately for children, other family members, and friends on a 6-point Likert scale ranging from 1 (three or more times a week) to 6 (less than once a year or never). Responses were reverse-coded to construct a sum score (range: 4–36), with higher values indicating more contacts with the social network. Respondents who skipped or did not answer more than two questions on frequency of contact were assigned missing scores. Although contacts by email or social media were asked in the HRS, they were not included in the sum score because relatively few adults age 65 + contact their social network via these methods [39]. Finally, the number of close relationships was measured as the sum of close relationships with children, family members, and friends (median = 8; Interquartile range (IQR) = 6).

***Neighborhood Social Cohesion.*** Neighborhood social cohesion was measured by self-report in the leave-behind survey. Respondents were asked to report on the conditions of the geographical area within a 20-minute walk of their primary residence. It assessed the respondents’ degree of agreement (on a scale of 0 to 6, with 6 being the strongest agreement) to the following four statements: *(1) I really feel part of this area*,* (2) Most people in this area can be trusted*,* (3) Most people in this area are friendly*, and *(4) If you were in trouble*,* there are lots of people in this area who would help you.* An index of neighborhood social cohesion was created by averaging scores across the four items (range: 0–6, median: 4.5, IQR: 2.5); a score of “missing” was assigned when three or all four responses were missing. Higher scores on the neighborhood social cohesion index indicated higher degrees of neighborhood social cohesion. In addition to this index, the analysis also included two binary indicators (yes/no) of (1) whether the respondents had *good friends* and (2) whether they had *relatives* in their neighborhood.

### Covariates

Demographic characteristics included age (years), gender (male, female), race/ethnicity (non-Hispanic White, non-Hispanic Black, Hispanic, other), education (less than high school, high school or equivalence, some college, college or above), marital status (never married, married/partnered, divorced/separated, widowed), household income (quartiles based on the main sample in USD: ≤$20,247, >$20,247–38,592, >$38,592–72,292, >$72,292) and total wealth (quartiles based on the main sample in USD: ≤$50,000, >$50,000–210,000, >$210,000–649,000, >$649,000). Health-related covariates included smoking (ever/never), alcohol use (ever/never), presence (vs. absence) of any physical comorbidities (among hypertension, diabetes, cancer, chronic lung disease, heart attack, stroke, and arthritis), and presence (vs. absence) of any basic activities of daily living limitations (among dressing, bathing, eating, toileting, walking/transferring).

### Analyses

Initially, characteristics of the main sample and the leave-behind subsamples were described using Student’s t-tests for continuous variables and chi-squared tests for categorical variables. Next, multivariable logistic regression models were fitted on the main sample to examine the associations between nursing home expectations (fitted as a continuous variable) and three outcomes, adjusting for demographic and health characteristics. Non-linearity of the expectations-mental health association was explored through quadratic terms but was not statistically significant (results not shown). Additional models were fit operationalizing expectations as a categorical variable, which overall produced similar results to the continuous measure. Finally, the potential moderating effect of social connectedness (social network characteristics and neighborhood social cohesion) were examined in the leave-behind sample using interaction terms (e.g., expectations*network diversity). Due to collinearity, interaction terms between expectations and each measure of social connectedness were examined in separate regression models.

Analyses were conducted using StataSE version 16.1 (StataCorp, College Station, TX) and survey procedures were used to account for the complex sampling design.

## Results

Table 1 shows the characteristics of older adults aged 65 + in the main and two leave-behind subsamples at the 2018 interview wave. Mean age of the main sample was 73.9 years; the majority was female (55%) and non-Hispanic White (80.5%). Approximately half reported at least one physical comorbidity, and one-fifth had at least one limitation in activities of daily living.

**Table 1:** Characteristics of older adults aged 65 + in main and Leave-Behind subset samples, 2018 health and retirement study

Characteristics	Main Sample	Social Network Leave-Behind Subset	Neighborhood Social Cohesion Leave-Behind Subset
	(*N* = 7897)	(*N* = 3034)	(*N* = 2577)
*Demographics*			
Age in years (mean ± SD)	73.9 ± 0.1	74.0 ± 0.2	73.8 ± 0.2
Sex, N (weighted %)			
Female	4664 (55.4)	1,776 (55.4)	1532 (56.4)
Race/ethnicity, N (weighted %)			
Non-Hispanic White	5385 (80.5)	2238 (81.6)	1932 (82.8)
Non-Hispanic Black	1326 (8.7)	432 (8.3)	359 (8.1)
Hispanic, regardless of race	973 (8.0)	286 (7.5)	224 (6.9)
Other	213 (2.8)	78 (2.5)	62 (2.3)
Education, N (weighted %)			
Less than high school	1288 (11.3)	383 (11.3)	304 (10.2)
High school or equivalence	2638 (31.6)	1047 (32.2)	883 (31.8)
Some college	1947 (25.5)	765 (24.7)	666 (25.3)
College or above	2024 (31.6)	839 (31.9)	724 (32.8)
Marital status, N (weighted %)			
Never married	275 (4.5)	78 (3.6)	75 (4.0)
Married/partnered	4532 (62.0)	1856 (63.4)	1587 (64.0)
Divorced/separated	1029 (12.6)	359 (12.3)	296 (11.8)
Widowed	2061 (21.0)	741 (20.9)	619 (20.2)
Household income, N (weighted %)			
US$0–20,247	1976 (19.5)	609 (17.8)	501 (16.8)
>US$20,247 − 38,592	1973 (22.3)	744 (22.5)	631 (22.5)
>US$38,592 − 72,292	1974 (26.3)	888 (29.5)	749 (29.4)
>US$72,292	1974 (31.8)	793 (30.3)	696 (31.3)
Total wealth, N (weighted %)			
≤US$50,000	1992 (20.1)	616 (17.5)	492 (16.3)
>US$50,000–210,000	1968 (22.8)	727 (23.0)	598 (21.9)
>US$210,00–649,000	1964 (25.8)	814 (26.5)	709 (27.3)
>US$649,000	1973 (31.4)	877 (33.0)	778 (34.5)
* Nursing Home Expectations*			
Percent chance of moving into nursing home in the next 5 years (mean ± SE)	15.3 ± 0.3	15.6 ± 0.5	15.6 ± 0.6
Categories of expectations, N (weighted %)			
0%	3277 (38.9)	1150 (37.0)	949 (35.5)
1–10%	1990 (28.8)	845 (30.2)	742 (31.5)
11–40%	1317 (17.9)	556 (18.3)	477 (18.7)
41–60%	947 (10.8)	351 (10.7)	300 (10.9)
61–100%	366 (3.7)	132 (3.8)	109 (3.5)
*Health Characteristics*			
Ever smoke, N (weighted %)	4273 (53.8)	1598 (52.6)	1331 (51.1)
Ever drink alcohol, N (weighted %)	4144 (56.6)	1656 (57.2)	1438 (58.3)
At least one chronic condition (hypertension, diabetes, cancer, chronic lung disease, heart attack, stroke, arthritis), N (weighted %)	3788 (50.9)	1560 (52.0)	1344 (52.8)
At least one ADL limitation (among dressing, bathing, eating, toileting, walking/transferring), N (weighted %)	1536 (16.9)	508 (18.0)	414 (17.1)
*Social Network Characteristics* ^a^			
Social network diversity, (mean ± SE)	-	3.2 ± 0.02	-
Contact with social network, (mean ± SE)	-	22.5 ± 0.1	-
Number of close relationships, (mean ± SE)	-	9.1 ± 0.2	-
*Neighborhood Social Cohesion Characteristics* ^b^			
Neighborhood social cohesion, (mean ± SE)	-	-	4.57 ± 0.03
Having relatives in neighborhood, N (weighted %)	-	-	715 (27.0)
Having good friends in neighborhood, N (weighted %)	-	-	1329 (50.2)
*Mental Health Outcomes*			
Elevated depressive symptoms, N (weighted %)	586 (7.5)	222 (7.5)	178 (7.0)
Major depressive episode, N (weighted %)	460 (5.8)	180 (5.9)	143 (5.6)
Passive suicidal ideation, N (weighted %)	396 (4.9)	143 (4.7)	111 (4.4)

ADL = activity of daily living. SE = Standard Error. Range of social network diversity (0–4), frequency of contact with social network (4–36), number of close relationships (0-139), and neighborhood social cohesion (0–6)

^a^ Statistics generated using social network leave-behind subsample (*N* = 3,034)

^b^ Statistics generated using social cohesion leave-behind subsample (*N* = 2,577)

Table 2 shows the characteristics of older adults aged 65 + in the main samples by categories of nursing home expectations. The majority of respondents reported low expectations of moving into a nursing home in the next 5 years (Supplementary Fig. 2). Overall, approximately two-thirds of respondents reported expectations equal to or below 10%, and nearly one fifth reported expectations between 11% and 40%. Compared to those with expectations of 0%, respondents who reported expectations between 1 and 40% were more likely to be male, wealthier, and have any physical comorbidities; they were less likely to have at least one ADL limitation. In contrast, respondents who reported expectations above 40% were older, more likely to be female, have at least one ADL limitation, and have poorer mental health than those with expectations less than 40%.

**Table 2:** Characteristics of the main sample by categories of nursing home expectations, 2018 health and retirement study (*N* = 7,897)

Characteristics	Nursing Home Expectation Category					
	0%	1–10%	11–40%	41–60%	61–100%	*p*-value ^a^
	(*N* = 3277)	(*N* = 1990)	(*N* = 1317)	(*N* = 947)	(*N* = 366)	
*Demographics*						
Age in years (mean ± SD)	73.6 ± 0.18	72.3 ± 0.20	73.9 ± 0.22	76.9 ± 0.37	80.3 ± 0.66	< 0.0001*
Sex, N (weighted %)						
Female	1978 (56.9)	1087 (50.9)	753 (53.6)	586 (59.7)	260 (71.3)	0.0002*
Race/ethnicity, N (weighted %)						
Non-Hispanic White	1900 (73.2)	1491 (85.4)	1049 (87.7)	696 (82.7)	249 (78.8)	
Non-Hispanic Black	737 (12.1)	268 (6.3)	132 (5.2)	135 (8.5)	54 (8.1)	< 0.0001*
Hispanic, regardless of race	537 (11.1)	186 (5.8)	111 (4.9)	91 (6.7)	48 (10.2)	
Other	103 (3.6)	45 (2.4)	25 (2.2)	25 (2.1)	15 (2.9)	
Education, N (weighted %)						
Less than high school	751 (17.0)	183 (5.6)	119 (5.6)	149 (12.6)	86 (18.2)	
High school or equivalence	1131 (34.4)	569 (25.0)	440 (30.7)	362 (37.9)	136 (39.7)	< 0.0001*
Some college	829 (26.9)	515 (25.6)	313 (24.2)	213 (24.0)	77 (21.6)	
College or above	566 (21.7)	723 (43.8)	445 (39.5)	223 (25.5)	67 (20.5)	
Marital status, N (weighted %)						
Never married	114 (4.3)	71 (4.7)	46 (4.9)	33 (3.5)	11 (5.4)	
Married/partnered	1753 (57.0)	1321 (71.1)	824 (65.7)	487 (56.4)	147 (41.9)	< 0.0001*
Divorced/separated	506 (15.5)	222 (10.2)	137 (10.6)	122 (12.7)	42 (9.0)	
Widowed	904 (23.2)	376 (14.0)	310 (18.8)	305 (27.4)	166 (43.8)	
Household income, N (weighted %)						
US$0–20,247	1030 (24.4)	325 (12.1)	239 (15.4)	253 (23.4)	129 (34.5)	
>US$20,247 − 38,592	894 (25.8)	411 (17.6)	311 (20.2)	256 (25.2)	101 (24.4)	< 0.0001*
>US$38,592 − 72,292	724 (25.0)	585 (28.5)	352 (26.3)	236 (26.6)	77 (23.0)	
>US$72,292	629 (24.8)	669 (41.8)	415 (38.1)	202 (24.9)	59 (18.1)	
Total wealth, N (weighted %)						
≤US$50,000	1117 (27.8)	325 (12.2)	205 (12.6)	224 (21.5)	121 (31.9)	
>US$50,000–210,000	891 (26.3)	440 (18.6)	297 (20.0)	250 (25.1)	90 (24.8)	< 0.0001*
>US$210,00–649,000	693 (21.5)	552 (28.9)	365 (28.6)	264 (28.4)	90 (25.1)	
>US$649,000	576 (24.4)	673 (40.4)	450 (38.9)	209 (25.1)	65 (18.3)	
*Health Characteristics*						
Ever smoke, N (weighted %)	1871 (58.1)	1041 (50.0)	677 (50.7)	504 (54.5)	180 (51.5)	0.025*
Ever drink alcohol, N (weighted %)	1569 (51.6)	1212 (64.5)	750 (61.0)	456 (49.3)	157 (47.5)	< 0.0001*
At least one chronic condition (hypertension, diabetes, cancer, chronic lung disease, heart attack, stroke, arthritis), N (weighted %)	1450 (46.7)	1089 (56.7)	704 (55.4)	413 (47.4)	132 (37.5)	0.001*
At least one ADL limitation (dressing, bathing, eating, toileting, walking/transferring), N (weighted %)	694 (19.0)	257 (10.4)	200 (13.6)	226 (23.4)	159 (43.1)	< 0.0001*
*Social Network Characteristics* ^b^						
Social network diversity, (mean ± SE)	3.1 ± 0.03	3.4 ± 0.03	3.2 ± 0.05	3.1 ± 0.04	2.9 ± 0.1	0.027*
Contact with social network, (mean ± SE)	22.1 ± 0.2	22.3 ± 0.3	23.1 ± 0.2	23.0 ± 0.5	23.5 ± 0.6	0.024*
Number of close relationships, (mean ± SE)	9.4 ± 0.3	8.4 ± 0.2	8.8 ± 0.4	10.1 ± 0.5	9.0 ± 0.6	0.002*
*Neighborhood Social Cohesion Characteristics* ^c^						
Neighborhood social cohesion, (mean ± SE)	4.5 ± 0.05	4.7 ± 0.05	4.7 ± 0.06	4.4 ± 0.08	4.3 ± 0.2	0.009*
Having relatives in neighborhood, N (weighted %)	296 (30.9)	185 (24.2)	124 (24.9)	78 (26.1)	32 (26.1)	0.36
Having good friends in neighborhood, N (weighted %)	491 (49.7)	379 (51.0)	237 (48.9)	155 (49.9)	67 (56.5)	0.65
*Mental Health Outcomes*						
Elevated depressive symptoms, N (weighted %)	256 (7.8)	125 (6.0)	93 (7.4)	73 (8.5)	39 (12.5)	0.007*
Major depressive episode, N (weighted %)	201 (5.9)	94 (4.5)	77 (6.0)	57 (7.0)	31 (10.2)	0.02*
Passive suicidal ideation, N (weighted %)	169 (4.8)	81 (3.8)	66 (4.9)	48 (5.7)	33 (11.0)	0.016*

ADL = activity of daily living

^a^ Student’s t-tests were used for continuous variables, chi-squared tests were used for categorical variables

^b^ Statistics generated using social network leave-behind subsample (*N* = 3,034)

^c^ Statistics generated using social cohesion leave-behind subsample (*N* = 2,577)

*p < 0.05

**Fig. 1:**
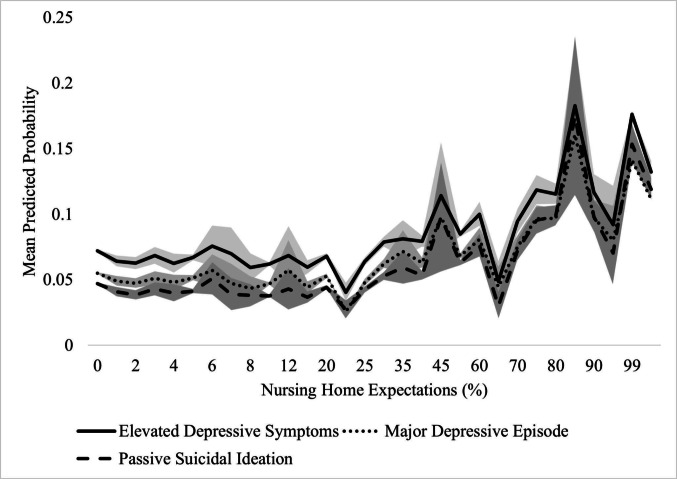
Predicted Probability of Mental Health Outcomes as a Function of Nursing Home Expectations (*N* = 7,897) (Health and Retirement Study, 2018)

As shown in Table 3, higher nursing home expectations were associated with higher odds of elevated depressive symptoms (adjusted odds ratio (AOR): 1.06, 95% CI: 1.01, 1.11), MDE (AOR: 1.08, 95% CI: 1.02, 1.15), and PSI (AOR: 1.10, 95% CI: 1.04, 1.17) for every 10% increase in nursing home expectations. Consistent with these results, Fig. 1 shows that higher nursing home expectations were associated with higher predicted probability of all three outcomes, with the predicted probabilities for MDE and PSI converging for expectations greater than 60%.

**Table 3:** Adjusted associations between nursing home expectations and mental health outcomes in main sample (*N* = 7,897) (Health and retirement study, 2018)

Nursing home expectations	Adjusted Odd Ratios (95% Confidence Interval) ^a^		
	Elevated Depressive Symptoms	Major depressive episode	Passive suicidal ideation
Continuous, 0–10	1.06 (1.01, 1.11)*	1.08 (1.02, 1.15)*	1.10 (1.04, 1.17)*
Model log likelihood, unweighted ^b^	*−1919.6*	*−1596.5*	*−1431.7*
Categorical			
0%	ref	ref	ref
1–10%	0.94 (0.68, 1.29)	0.96 (0.69, 1.32)	1.08 (0.70, 1.67)
11–40%	1.14 (0.83, 1.56)	1.27 (0.90, 1.78)	1.32 (0.94, 1.85)
41–60%	1.25 (0.86, 1.81)	1.40 (0.88, 2.23)	1.37 (0.81, 2.32)
61–100%	1.62 (1.05, 2.51)*	1.84 (1.06, 3.18)*	2.32 (1.36, 3.94)*

^a^ All models adjusted for age, gender, race/ethnicity, education, marital status, household income, total wealth, smoking status, alcohol use, physical comorbidities, and activities of daily living

^b^ The maximum likelihood assumption is violated when using survey weights in Stata, and thus unweighted log likelihood is used

*p < 0.05

Table 4 shows the adjusted odds ratios from the moderation analyses by social connectedness. In all analyses, nursing home expectations was not significantly associated with depressive outcomes after adjusting for social connectedness measures and their interaction terms, except for having relatives living in the neighborhood. Specifically, even after accounting for having relatives living in the neighborhood and its interaction with nursing home expectations, higher nursing home expectations were associated with higher odds of elevated depressive symptoms (AOR: 1.10, 95% CI: 1.01, 1.18), MDE (AOR: 1.11, 95% CI: 1.02, 1.20), and PSI (AOR: 1.10, 95% CI: 1.01, 1.20). In general, social connectedness and all the interaction terms were not significantly associated with depressive outcomes, with the exception of the interaction between nursing home expectations and having good friends living in the respondent’s neighborhood. Compared to those without, respondents with good friends in the neighborhood had greater odds ratio of elevated depressive symptoms (ratio of OR = 1.21 (95% CI = 1.04, 1.42)) and PSI (ratio of OR = 1.25 (95% CI = 1.02, 1.52)).

**Table 4:** Adjusted odds ratios for social connectedness measures and the interaction between social connectedness and nursing home expectations on mental health outcomes in Leave-Behind subsets, 2018 health and retirement study

	Adjusted Odd Ratios (95% Confidence Interval) ^a^		
	Elevated Depressive Symptoms	Major Depressive Episode	Passive Suicidal Ideation
*Interaction: Social network x Nursing home expectations* ^*b*^			
Nursing home expectations	1.10 (0.87, 1.40)	1.09 (0.83, 1.44)	1.06 (0.82, 1.37)
Diversity	1.13 (0.81, 1.57)	1.10 (0.80, 1.52)	0.92 (0.68, 1.25)
Diversity x Expectations	0.99 (0.91, 1.07)	1.00 (0.91, 1.09)	1.01 (0.93, 1.10)
Nursing home expectations	1.02 (0.81, 1.28)	1.02 (0.81, 1.28)	0.97 (0.77, 1.21)
Frequency of contact	0.98 (0.94, 1.01)	0.98 (0.95, 1.01)	0.97 (0.93, 1.01)
Frequency of contact x Expectations	1.00 (0.99, 1.01)	1.00 (0.99, 1.01)	1.01 (1.00, 1.02)
Nursing home expectations	1.03 (0.92, 1.15)	1.04 (0.93, 1.17)	1.16 (1.03, 1.31)
Number of close relationships	0.98 (0.95, 1.01)	0.99 (0.96, 1.02)	0.98 (0.94, 1.01)
Number of close relationships x Expectations	1.00 (0.99, 1.01)	1.00 (0.99, 1.01)	0.99 (0.98, 1.00)
*Interaction: Neighborhood social cohesion x Nursing home expectations* ^*c*^			
Nursing home expectations	0.99 (0.82, 1.21)	1.07 (0.86, 1.34)	1.09 (0.85, 1.40)
Neighborhood social cohesion index	0.78 (0.66, 0.93)*	0.85 (0.69, 1.04)	0.91 (0.73, 1.13)
Neighborhood social cohesion index x Expectations	1.01 (0.97, 1.06)	1.00 (0.94, 1.06)	1.00 (0.94, 1.06)
Nursing home expectations	0.95 (0.84, 1.09)	0.99 (0.86, 1.14)	0.96 (0.82, 1.12)
Having good friends live in respondent’s neighborhood	0.64 (0.36, 1.12)	0.93 (0.49, 1.77)	0.77 (0.43, 1.35)
Having good friends live in respondent’s neighborhood x Expectations	1.21 (1.04, 1.42)*	1.16 (0.97, 1.38)	1.25 (1.02, 1.52)*
Nursing home expectations	1.10 (1.01, 1.18)*	1.11 (1.02, 1.20)*	1.10 (1.01, 1.20)*
Having relatives living in respondent’s neighborhood	1.02 (0.64, 1.64)	1.05 (0.64, 1.72)	0.75 (0.42, 1.34)
Having relatives living in respondent’s neighborhood x Nursing home expectations	0.78 (0.61, 1.02)	0.82 (0.64, 1.04)	0.91 (0.71, 1.16)

^a^ Each social connectedness variable*expectations interaction term was tested in a separate model. Models included nursing home expectation, social connectedness variable of interest, interaction term between nursing home expectation and social connectedness variable of interest, age, gender, race/ethnicity, education, marital status, household income, total wealth, smoking status, alcohol use, physical comorbidities, and activities of daily living

^b^ Models used social network leave-behind subsample (*N* = 3,034)

^c^ Models used social cohesion leave-behind subsample (*N* = 2,577)

*p < 0.05

## Discussion

This cross-sectional study examined the relationship between expectations regarding moving into a nursing home in the next five years and depressive symptomatology among older US adults. The primary findings of the study are twofold: First, older adults who anticipated a higher likelihood of moving into a nursing home reported poorer mental health, as evidenced by significantly higher odds of elevated depressive symptoms, MDE, and PSI. Second, the association between nursing home expectations and depressive symptomatology generally did not vary by social connectedness (social network characteristics or neighborhood social cohesion), with one exception: those reporting the presence of good friends living in the neighborhood showed an elevated association. These findings emphasize that older adults who are anticipating moving into residential care have substantial mental health needs, and thus efforts to assist older adults and their families need to begin long before the actual relocation into residential care takes place. Moreover, it may be fruitful to consider neighborhood social life characteristics, such as presence of close friends, to support older adults during care transitions, whether those transitions involve relocation to residential LTC or home modifications to support “aging in place.”

The finding that nursing home expectations were positively associated with depressive symptomatology is consistent with prior literature linking expectations of negatively-perceived events to constructs related to depression such as hopelessness. Indeed, previous research has shown that hopelessness [40] and lacking a sense of purpose or meaning of life [41] are associated with poor mental health. Moreover, low tolerance for uncertainty may accentuate distress surrounding the possibility of needing nursing home care via depressive symptoms [42]. In this study, respondents who expressed more uncertainty about their likelihood of moving into nursing homes (i.e., reporting expectations of 1–40%) were less likely to have ADL limitations than those reporting *absolutely no chance* of this occurring (i.e., expectation of 0%). This suggests the negative beliefs about the uncertainty during the transition into nursing homes, in spite of the actual health needs, may contribute to depressive symptomatology. While research on the mediating role of intolerance of uncertainty is largely limited to younger adults [43], future research in this area should examine older populations and types of transitions (e.g., becoming widowed, moving into a nursing home) that this group is more likely to experience compared to younger groups.

Findings from the moderation analyses by social connectedness are twofold. First, we found that nursing home expectations was generally no longer significantly associated with depressive symptomatology after adjusting for social connectedness measures. This finding suggests that the relationship between nursing home expectations and mental health could be explained by their correlations with social connectedness. Indeed, previous research demonstrates that social connectedness is associated with better mental health among nursing home residents [44] and older adults transitioning to long-term care settings [4, 45]. It is worth noting that nursing home expectations remained positively associated with depressive symptomatology, even after accounting for having relatives living in respondent’s neighborhood. Given that older adults often receive caregiving help from their spouses or children [46], it is possible that relatives are not involved in caregiving responsibilities and/or the decisions relating to LTC transitions, thus may not influence the likelihood of transitioning into nursing homes and mental health.

Second, we did not find evidence suggesting that social connectedness measures, generally speaking, were associated with depressive symptomatology, nor that they modified the association between nursing home expectations and mental health. There are a few potential explanations for this finding. First, the measures of social network and neighborhood social cohesion examined in this study may not adequately capture aspects of social life (e.g., quality of social relationships, sense of personal identity [47], or resilience [48]) that are most relevant to mental health. Indeed, prior work has argued that the quality of support has a stronger protective effect on mental health than social network structure and diversity [49, 50]. While neighborhood social cohesion is correlated with social participation [51] and improved mental health [52], it is possible that older adults who expect to move into nursing homes have mobility challenges that result in limited engagement with their neighbors or neighborhood organizations. If this is the case, then perceived neighborhood social cohesion may be unlikely to influence the association between nursing home expectations and mental health. Finally, the moderation analysis was limited to respondents who completed the leave-behind survey which has smaller sample sizes compared to the entire HRS and thus may be underpowered to detect modest effects.

Unexpectedly, although having good friends living in the neighborhood was not significantly associated with depressive symptomatology, there is suggestive evidence that it moderated the association between nursing home expectations and depressive symptomatology. In particular, the presence of good friends enhanced, rather than buffered, the association between nursing home expectations and elevated depressive symptoms and PSI. These findings suggest that, while living in close proximity to good friends may not, on its own, have a significant impact on mental health, older adults may experience anticipatory social loss stemming from leaving behind a close-knit social circle or community upon transition into LTC [53]. Indeed, Buckley and McCarthy (2009) found that nursing home residents expressed feeling disconnected from the outside world and reported difficulty in building new friendships within their nursing home community [54]. Additionally, physical distance between older adults’ prior residence and their nursing home may exacerbate feelings of social isolation as friends and family may not be able to visit frequently [55]. Collectively, these studies suggest that facets of social life such as quality of friendships, types of support, and physical proximity are relevant to the experience of moving into LTC, even if they do not necessarily operate through expectations. Future studies can examine these contextual factors to better elucidate the pathways linking close friendships to mental health in anticipation of, during, and after nursing home transitions.

Overall, the study findings highlight the need to support older adults during all parts of LTC transitions, including anticipation of moving into residential care. While there are existing interventions designed for implementation *after* older adults move into nursing homes and related settings [56], this study demonstrates that more programs are needed to help older adults navigate long-term care planning prior to such transitions [57]. For example, behavioral counseling can help older adults understand and navigate feelings of depression that may occur before, during, and after LTC transitions [58]. Psychosocial interventions focusing on enhancing social connections, either face-to-face or through the use of technology, are also warranted. A successful example is the social network application “Media Parcels” developed by Zaine et al. (2019), which promotes social connections among older adults via “wrapped” media messages containing photos or audio of shared memories [59]. Such efforts offer opportunities for social connections among older adults independent of their living scenario.

### Strengths and limitations

Findings should be interpreted in light of study limitations. First, this study only assessed expectations related to *nursing home* transitions. Therefore, findings may not be generalizable to expectations for other residential settings in later life, such as assisted living facilities or moving in with adult children. Second, the analytical sample was more likely to be white and wealthy compared to the full HRS, and thus caution is warranted when generalizing the findings to racial minority or lower income groups. Third, nursing home expectations may be shaped by the availability of community-based supports for “aging in place” (e.g., access to housekeeping services, transportation, proximity to goods and services) and older adults’ ability to *mobilize* help from their social networks, above and beyond the size and closeness of their networks [60]. However, these types of specific measures were not available in the data used for this study. Moreover, the study did not examine personality traits (e.g., locus of control) and assistance with ADLs/IADLs, which may also influence the expectations of transitioning into nursing homes and mental health. Fourth, social connectedness were assessed by self-report, and respondents with limited social networks may skip or inaccurately answer questions about social network characteristics due to social desirability bias. Fifth, while the HRS is a longitudinal cohort, this was a cross-sectional analysis that used data only from the 2018 wave, introducing a potential for reverse causation or simultaneity bias. This design decision was necessary given the dramatic impact of the COVID-19 pandemic on LTC settings [61]; we felt that including the 2020 and later data would be qualitatively non-comparable on our exposure of interest and inappropriate for our research question. We thus chose to examine only the most contemporaneous wave of data that was not impacted by the pandemic. It is critical that future studies examine the impact of the pandemic on the expectations and actual transitions into LTC. Finally, while prior work has examined the mental health benefits of “met” versus “unmet” expectations (e.g., expectations regarding future labor force status [62]), the notion of mental health benefits of “met” nursing home expectations is unlikely given that the majority of older adults prefer to age in place [63].

Despite limitations, this study has several strengths. To the best of our knowledge, this study is among the first to investigate the relationship between *expectations* of moving into a nursing home and mental health among *community-residing* older adults. By examining nursing home expectations in and of themselves, this study provides insights into how anticipation of the functional and health challenges that typically prompt a transition into residential care [63], including the symbolic meaning of declining independence [64], contribute to mental health. Additionally, the HRS is one of largest and most comprehensive surveys of older adults, which enhances the external validity of our findings.

## Conclusion

The study found that higher expectation of moving into a nursing home in the near future is associated with higher odds of depressive symptomatology even after accounting for health status and functional impairment. Beyond emphasizing the need to address the emotional distress that often accompanies residential care transitions, these findings point to potential opportunities for addressing the mental health needs of older adults in later life, regardless of whether they eventually move into LTC (e.g., through care and social services navigation support).

## Supplementary Information

Below is the link to the electronic supplementary material.

Supplementary Material 1

## Data Availability

The Health and Retirement Study data is publicly available at https://hrs.isr.umich.edu.

## References

[1] Hurd MD, Michaud P-C, Rohwedder S (2017) Distribution of lifetime nursing home use and of out-of-pocket spending. Proc Natl Acad Sci U S A 114(37):9838–9842. 10.1073/pnas.1700618114 28847934 10.1073/pnas.1700618114 PMC5603996

[2] Toth M et al Understanding the Characteristics of Older Adults in Different Residential Settings: Data Sources and Trends, United States Department of Health and Human Services, Office of the Assistant Secretary for Planning and Evaluation, Behavioral Health, Disability and Aging Policy, Washington, D.C., Oct. 2020. Accessed: Feb. 28, 2025. [Online]. Available: http://aspe.hhs.gov/reports/understanding-characteristics-older-adults-different-residential-settings-data-sources-trends

[3] Costlow K, Parmelee PA (2020) The impact of relocation stress on cognitively impaired and cognitively unimpaired long-term care residents. Aging Ment Health 24(10):1589–1595. 10.1080/13607863.2019.1660855 31468988 10.1080/13607863.2019.1660855 PMC7048638

[4] Brownie S, Horstmanshof L, Garbutt R (2014) Factors that impact residents’ transition and psychological adjustment to long-term aged care: a systematic literature review. Int J Nurs Stud 51(12):1654–1666. 10.1016/j.ijnurstu.2014.04.011 24813582 10.1016/j.ijnurstu.2014.04.011

[5] Long Term Services & Supports| Medicaid. Accessed: Feb. 28, 2025. [Online]. Available: https://www.medicaid.gov/medicaid/long-term-services-supports/index.html

[6] Mayo CD, Kenny R, Scarapicchia V, Ohlhauser L, Syme R, Gawryluk JR (2021) Aging in Place: Challenges of Older Adults with Self-Reported Cognitive Decline, *Can. Geriatr. J.*, vol. 24, no. 2, pp. 138–143, Jun. 10.5770/cgj.24.456 10.5770/cgj.24.456 PMC8137463 34079607

[7] Tang F, Lee Y (2011) Social support networks and expectations for aging in place and moving. Res Aging 33(4):444–464. 10.1177/0164027511400631

[8] Lindrooth RC, Hoerger TJ, Norton EC (2000) Expectations among the elderly about nursing home entry. Health Serv Res 35(5 Pt 2):1181–1202 11130816 PMC1089169

[9] Taylor DH, Osterman J, Acuff SW, Østbye T (Jun.2005) Do seniors understand their risk of moving to a nursing home?? Health Serv Res 40(3):811–828. 10.1111/j.1475-6773.2005.00386.x 15960692 10.1111/j.1475-6773.2005.00386.x PMC1361169

[10] Samsi K, Cole L, Manthorpe J (2022) ‘The time has come’: reflections on the ‘tipping point’ in deciding on a care home move. Aging Ment Health 26(9):1855–1861. 10.1080/13607863.2021.1947963 34278912 10.1080/13607863.2021.1947963

[11] Rustad EC, Furnes B, Cronfalk BS, Dysvik E (May 2016) Older patients’ experiences during care transition. Patient Prefer Adherence 10:769–779. 10.2147/PPA.S97570 10.2147/PPA.S97570 PMC4869594 27274204

[12] Travers JL, Hirschman KB, Naylor MD (2022) Older Adults’ Goals and Expectations When Using Long-Term Services and Supports, *J. Appl. Gerontol. Off. J. South. Gerontol. Soc.*, vol. 41, no. 3, pp. 709–717, Mar. 10.1177/07334648211033671 10.1177/07334648211033671 PMC8792093 34315240

[13] Thein NW, D’Souza G, Sheehan B (Feb. 2011) Expectations and experience of moving to a care home: perceptions of older people with dementia. Dementia 10(1):7–18. 10.1177/1471301210392971

[14] De Poli C, Wittenberg R, Rehill A, Stevens M, Brimblecombe N (2025) I never planned for it’—Exploration of expectations about caring for older parents. Soc Policy Adm 59(1):37–56. 10.1111/spol.13030

[15] Aberg KC, Toren I, Paz R (2022) A neural and behavioral trade-off between value and uncertainty underlies exploratory decisions in normative anxiety. Mol Psychiatry 27(3):1573–1587. 10.1038/s41380-021-01363-z 34725456 10.1038/s41380-021-01363-z

[16] Beck AT, Weissman A, Lester D, Trexler L (1974) The measurement of pessimism: the hopelessness scale. J Consult Clin Psychol 42(6):861–865. 10.1037/h0037562 4436473 10.1037/h0037562

[17] Giner L, Blasco-Fontecilla H, De La Vega D, Courtet P (2016) Cognitive, emotional, temperament, and personality trait correlates of suicidal behavior. Curr Psychiatry Rep 18(11):102. 10.1007/s11920-016-0742-x 27726066 10.1007/s11920-016-0742-x

[18] Assari S, Lankarani MM (May 2016) Depressive symptoms are associated with more hopelessness among white than black older adults. Front Public Health 4:82. 10.3389/fpubh.2016.00082 10.3389/fpubh.2016.00082 PMC4854870 27200335

[19] MacLeod AK, O’Connor RC (2018) Positive future–thinking, well–being, and mental health. In Oettingen G, Sevincer AT, and Gollwitzer P (Eds.), The psychology of thinking about the future. The Guilford Press, New York, NY, US, pp 199–213

[20] Thimm JC, Holte A, Brennen T, Wang CEA (Jul. 2013) Hope and expectancies for future events in depression. Front Psychol 4:470. 10.3389/fpsyg.2013.00470 10.3389/fpsyg.2013.00470 PMC3721024 23898314

[21] Loebel JP, Loebel JS, Dager SR, Centerwall BS, Reay DT (Apr. 1991) Anticipation of nursing home placement May be a precipitant of suicide among the elderly. J Am Geriatr Soc 39(4):407–408. 10.1111/j.1532-5415.1991.tb02910.x 10.1111/j.1532-5415.1991.tb02910.x 2010593

[22] Mezuk B, Rock A, Lohman MC, Choi M (Dec. 2014) Suicide risk in long-term care facilities: a systematic review. Int J Geriatr Psychiatry 29(12):1198–1211. 10.1002/gps.4142 10.1002/gps.4142 PMC4232590 24854089

[23] Mezuk B, Ko TM, Kalesnikava VA, Jurgens D (2019) Suicide among older adults living in or transitioning to residential long-term care, 2003 to 2015. JAMA Netw Open 2(6):e195627. 10.1001/jamanetworkopen.2019.5627 31199445 10.1001/jamanetworkopen.2019.5627 PMC6575144

[24] Podgorski CA, Langford L, Pearson JL, Conwell Y (2010) Suicide prevention for older adults in residential communities: implications for policy and practice. PLoS Med 7(5):e1000254. 10.1371/journal.pmed.1000254 20502523 10.1371/journal.pmed.1000254 PMC2872646

[25] Luong G, Charles ST, Fingerman KL (Feb. 2011) Better with age: social relationships across adulthood. J Soc Pers Relatsh 28(1):9–23. 10.1177/0265407510391362 10.1177/0265407510391362 PMC3291125 22389547

[26] Wickramaratne PJ et al (2022) Social connectedness as a determinant of mental health: a scoping review. PLoS One 17(10):e0275004. 10.1371/journal.pone.0275004 36228007 10.1371/journal.pone.0275004 PMC9560615

[27] Reiner A, Steinhoff P (2024) The association of social networks and depression in community-dwelling older adults: a systematic review. Syst Rev 13:161. 10.1186/s13643-024-02581-6 38902787 10.1186/s13643-024-02581-6 PMC11188217

[28] van Tilburg T (1998) Losing and gaining in old age: changes in personal network size and social support in a four-year longitudinal study. The Journals of Gerontology Series B: Psychological Sciences and Social Sciences 53(6):S313–S323. 10.1093/geronb/53B.6.S313 9826973 10.1093/geronb/53b.6.s313

[29] Chan N, Anstey KJ, Windsor TD, Luszcz MA (2011) Disability and depressive symptoms in later life: the stress-buffering role of informal and formal support. Gerontology 57(2):180–189. 10.1159/000314158 20424429 10.1159/000314158 PMC3214839

[30] Shulyaev K, Spielberg Y, Gur-Yaish N, Zisberg A (2024) Family support during hospitalization buffers depressive symptoms among independent older adults. Clin Gerontol 47(2):341–351. 10.1080/07317115.2023.2236097 37493087 10.1080/07317115.2023.2236097

[31] Kim ES, Chen Y, Kawachi I, VanderWeele TJ (Nov.2020) Perceived neighborhood social cohesion and subsequent health and well-being in older adults: an outcome-wide longitudinal approach. Health Place 66:102420. 10.1016/j.healthplace.2020.102420 32905980 10.1016/j.healthplace.2020.102420 PMC7686282

[32] Zeng D, Wu X Neighborhood collective efficacy in stressful events: The stress-buffering effect, *Soc. Sci. Med.* (1982), vol. 306, p. 115154, Aug. 2022, doi:, vol. 306, p. 115154, Aug. 2022. 10.1016/j.socscimed.2022.115154 10.1016/j.socscimed.2022.115154 35753169

[33] Choi YJ, Matz-Costa C (2018) Perceived neighborhood safety, social cohesion, and psychological health of older adults. Gerontologist 58(1):196–206. 10.1093/geront/gnw187 28082279 10.1093/geront/gnw187

[34] Seplaki CL, Goldman N, Weinstein M, Lin Y-H (2006) Before and after the 1999 Chi-Chi earthquake: traumatic events and depressive symptoms in an older population. Soc Sci Med 62(12):3121–3132. 10.1016/j.socscimed.2005.11.059 16423437 10.1016/j.socscimed.2005.11.059

[35] Chao S-F (2016) Outdoor activities and depressive symptoms in displaced older adults following natural disaster: community cohesion as mediator and moderator. Aging Ment Health 20(9):940–947. 10.1080/13607863.2015.1044940 25985261 10.1080/13607863.2015.1044940

[36] Sonnega A, Faul JD, Ofstedal MB, Langa KM, Phillips JW, Weir DR (Apr. 2014) Cohort profile: the health and retirement study (HRS). Int J Epidemiol 43(2):576–585. 10.1093/ije/dyu067 10.1093/ije/dyu067 PMC3997380 24671021

[37] Kessler RC, Andrews G, Mroczek D, Ustun B, Wittchen H-U (1998) The world health organization composite international diagnostic interview short-form (CIDI-SF). Int J Methods Psychiatr Res 7(4):171–185. 10.1002/mpr.47

[38] American Psychiatric Association (2022) Ed., *Diagnostic and Statistical Manual of Mental Disorders| Psychiatry Online*, 5th ed

[39] Rosentiel T (2010) Older Adults and Social Media, Pew Research Center, Aug. Accessed: Feb. 28, 2025. [Online]. Available: https://www.pewresearch.org/2010/08/27/older-adults-and-social-media/

[40] MacLeod AK, Tata P, Tyrer P, Schmidt U, Davidson K, Thompson S (2005) Hopelessness and positive and negative future thinking in parasuicide. Br J Clin Psychol 44(Pt 4):495–504. 10.1348/014466505X35704 16368029 10.1348/014466505X35704

[41] Sun F-K, Wu M-K, Yao Y, Chiang C-Y, Lu C-Y (2022) Meaning in life as a mediator of the associations among depression, hopelessness and suicidal ideation: a path analysis. J Psychiatr Ment Health Nurs 29(1):57–66. 10.1111/jpm.12739 33559221 10.1111/jpm.12739

[42] Saulnier KG, Allan NP, Raines AM, Schmidt NB (2019) Depression and intolerance of uncertainty: relations between uncertainty subfactors and depression dimensions. Psychiatry 82(1):72–79. 10.1080/00332747.2018.1560583 30730786 10.1080/00332747.2018.1560583

[43] Regnoli GM, Tiano G, De Rosa B (2024) Serial Mediation Models of Future Anxiety and Italian Young Adults Psychological Distress: The Role of Intolerance of Uncertainty and Non-Pathological Worry, *Eur. J. Investig. Health Psychol. Educ.*, vol. 14, no. 6, pp. 1834–1852, Jun. 10.3390/ejihpe14060121 10.3390/ejihpe14060121 PMC11202537 38921087

[44] Lim E et al (Nov. 2023) Health effects of social connectedness in older adults living in congregate long term care settings: A systematic review of quantitative and qualitative evidence. Int J Older People Nurs 18(6):e12577. 10.1111/opn.12577 10.1111/opn.12577 PMC10843483 37803996

[45] Zamanzadeh V, Rahmani A, Pakpour V, Chenoweth LL, Mohammadi E (Jun. 2017) Psychosocial changes following transition to an aged care home: qualitative findings from Iran. Int J Older People Nurs 12(2). 10.1111/opn.12130 10.1111/opn.12130 27709808

[46] Wolff JL, Cornman JC, Freedman VA (2025) The number of family caregivers helping older US adults increased from 18 million to 24 million, 2011–22. Health Aff. (Millwood) 44(2):187–195. 10.1377/hlthaff.2024.00978 39899774 10.1377/hlthaff.2024.00978 PMC11869104

[47] Praharso NF, Tear MJ, Cruwys T (Jan.2017) Stressful life transitions and wellbeing: a comparison of the stress buffering hypothesis and the social identity model of identity change. Psychiatry Res 247:265–275. 10.1016/j.psychres.2016.11.039 27936438 10.1016/j.psychres.2016.11.039

[48] Zhao X et al (2018) Loneliness and depression symptoms among the elderly in nursing homes: a moderated mediation model of resilience and social support. Psychiatry Res 268:143–151. 10.1016/j.psychres.2018.07.011 30025285 10.1016/j.psychres.2018.07.011

[49] Antonucci TC (2001) Social relations: an examination of social networks, social support, and sense of control. In Birren JE and Schaie KW (Eds.), Handbook of the psychology of aging, 5th edn. Academic, San Diego, CA, US, pp 427–453

[50] Fiori KL, Antonucci TC, Cortina KS (Jan. 2006) Social network typologies and mental health among older adults. J Gerontol B Psychol Sci Soc Sci 61(1):P25–32. 10.1093/geronb/61.1.p25 10.1093/geronb/61.1.p25 16399938

[51] Latham K, Clarke PJ, Disorder N (Jan. 2018) Perceived social cohesion, and social participation among older americans: findings from the National health & aging trends study. J Aging Health 30(1):3–26. 10.1177/0898264316665933 10.1177/0898264316665933 27566464

[52] Elliott J, Gale CR, Parsons S, Kuh D (Apr. 2014) Neighbourhood cohesion and mental wellbeing among older adults: A mixed methods approach. Soc Sci Med 107:44–51. 10.1016/j.socscimed.2014.02.027 10.1016/j.socscimed.2014.02.027 24602970

[53] Hvalvik S, Åse I, Reierson (Jan. 2011) Transition from self-supported to living: older people’s experiences. Int J Qual Stud Health Well-Being 6(4):7914. 10.3402/qhw.v6i4.7914 10.3402/qhw.v6i4.7914 PMC3228403 22135699

[54] Buckley C, McCarthy G (2009) An Exploration of Social Connectedness as Perceived by Older Adults in a Long-Term Care Setting in Ireland, *Geriatr. Nur. (Lond.)*, vol. 30, no. 6, pp. 390–396, Nov. 10.1016/j.gerinurse.2009.09.001 10.1016/j.gerinurse.2009.09.001 19963148

[55] Parmenter G, Cruickshank M, Hussain R (Feb.2012) The social lives of rural Australian nursing home residents. Ageing Soc 32(2):329–353. 10.1017/S0144686X11000304

[56] Groenvynck L et al (2022) Interventions to improve the transition from home to a nursing home: a scoping review. Gerontologist 62:e369–e383. 10.1093/geront/gnab036 33704485 10.1093/geront/gnab036 PMC9372886

[57] Ryan A, McKenna H (2013) ‘Familiarity’ as a key factor influencing rural family carers’ experience of the nursing home placement of an older relative: a qualitative study. BMC Health Serv Res 13:252. 10.1186/1472-6963-13-252 23822872 10.1186/1472-6963-13-252 PMC3704947

[58] Britton PC, Duberstein PR, Conner KR, Heisel MJ, Hirsch JK, Conwell Y (2008) Reasons for Living, Hopelessness, and Suicide Ideation Among Depressed Adults 50 Years or Older, *Am. J. Geriatr. Psychiatry Off. J. Am. Assoc. Geriatr. Psychiatry*, vol. 16, no. 9, pp. 736–741, Sep. 10.1097/JGP.0b013e31817b609a 10.1097/JGP.0b013e31817b609a PMC2763305 18757767

[59] Zaine I et al (Oct. 2019) Promoting social connection and deepening relations among older adults: design and qualitative evaluation of media parcels. J Med Internet Res 21(10):e14112. 10.2196/14112 10.2196/14112 PMC6797971 31584001

[60] Lehning AJ, Smith RJ, Dunkle RE (2015) Do Age-Friendly Characteristics Influence the Expectation to Age in Place? A Comparison of Low-Income and Higher Income Detroit Elders, *J. Appl. Gerontol. Off. J. South. Gerontol. Soc.*, vol. 34, no. 2, pp. 158–180, Mar. 10.1177/0733464813483210 10.1177/0733464813483210 PMC4640671 24652879

[61] Chen AT, Ryskina KL, Jung H-Y, Care L-T (2020) Residential Facilities, and COVID-19: An Overview of Federal and State Policy Responses, *J. Am. Med. Dir. Assoc.*, vol. 21, no. 9, pp. 1186–1190, Sep. 10.1016/j.jamda.2020.07.001 10.1016/j.jamda.2020.07.001 PMC7334928 32859298

[62] Abrams LR, Clarke PJ, Mehta NK (2022) Unmet expectations about work at age 62 and depressive symptoms. J Gerontol B Psychol Sci Soc Sci 77(3):615–625. 10.1093/geronb/gbab113 34173825 10.1093/geronb/gbab113

[63] Fausset CB, Kelly AJ, Rogers WA, Fisk AD (2011) Challenges to aging in place: understanding home maintenance difficulties. J Hous Elder 25(2):125–141. 10.1080/02763893.2011.571105 10.1080/02763893.2011.571105 PMC3209521 22072843

[64] AARP Livable Communities - AARP.org/ Livable - Inspiration and Information for Local Leaders, AARP. Accessed: Feb. 28, 2025. [Online]. Available: https://www.aarp.org/livable-communities/

