# Supplementary material for: Expectations regarding transitioning into long-term care, social connectedness, and mental health of older adults
Source: Soc Psychiatry Psychiatr Epidemiol. 2025 Aug 18;61(5):853–66. doi: 10.1007/s00127-025-02975-4 (PMC12439355; doi:10.1007/s00127-025-02975-4)

**Expectations regarding transitioning into long-term care, social connectedness, and mental health of older adults**

Annalise Lane, MPH^1,2^, Linh Dang, PhD^1^, Weidi Qin, PhD^3^, Sarah Burgard, PhD^4,5^, Briana Mezuk, PhD^1,5^

^1^ Center for Social Epidemiology and Population Health, Department of Epidemiology, University of Michigan School of Public Health, Ann Arbor, Michigan, USA.

^2^ Department of Health Management and Policy, University of Michigan School of Public Health, Ann Arbor, Michigan, USA.

^3^ Sandra Rosenbaum School of Social Work, University of Wisconsin–Madison, Madison, Wisconsin, USA.

^4^ Department of Sociology, University of Michigan, Ann Arbor, Michigan, USA.

^5^ Institute for Social Research, University of Michigan, Ann Arbor, Michigan, USA.

For submission to the Special Issue “Ageing and mental health” in *Social Psychiatry and Psychiatric Epidemiology*

Corresponding Author: Briana Mezuk, PhD

University of Michigan School of Public Health

1415 Washington Heights

Ann Arbor, MI 48109, USA

E-mail: bmezuk@umich.edu

**Supplementary Table 1**. Characteristics of Older Adults Included and Excluded from Main Sample, 2018 Health and Retirement Study

| **Characteristics** | **Total 2018 HRS Sample** | **Main Analytical Sample** | **Excluded Respondents** |  |
| --- | --- | --- | --- | --- |
|  | (N=17146) | (N=7897) | (N=9249) |  |
| ***Demographics*** |  |  |  |  |
| Age in years (mean ± SD) | 66.3 ± 0.20 | 73.9 ± 0.14 | 60.1 ± 0.14 | <0.0001* |
| Sex, N (weighted %) |  |  |  |  |
| Female | 10115 (53.7) | 4664 (55.4) | 5451 (52.2) | 0.87 |
| Race/ethnicity, N (weighted %) |  |  |  |  |
| Non-Hispanic White | 9749 (73.8) | 5385 (80.5) | 4364 (68.2) |  |
| Non-Hispanic Black | 3696 (10.5) | 1326 (8.7) | 2370 (12.0) | <0.0001* |
| Hispanic, regardless of race | 2843 (10.3) | 973 (8.0) | 1870 (12.3) |  |
| Other | 853 (5.4) | 213 (2.8) | 640 (7.5) |  |
| Education, N (weighted %) |  |  |  |  |
| Less than high school | 2856 (11.2) | 1288 (11.3) | 1568 (11.2) |  |
| High school or equivalence | 5426 (29.9) | 2638 (31.6) | 2788 (28.5) | <0.0001* |
| Some college | 4576 (27.5) | 1947 (25.5) | 2629 (29.2) |  |
| College or above | 4285 (31.4) | 2024 (31.6) | 2261 (31.1) |  |
| Marital status, N (weighted %) |  |  |  |  |
| Never married | 1109 (7.4) | 275 (4.5) | 834 (9.7) |  |
| Married/partnered | 10462 (65.1) | 4532 (62.0) | 5930 (67.7) | <0.0001* |
| Divorced/separated | 2689 (14.8) | 1029 (12.6) | 1660 (16.7) |  |
| Widowed | 2860 (12.7) | 2061 (21.0) | 799 (5.9) |  |
| Household income, N (weighted %) | | | |  |
| US$0 - 20,247 | 4481 (19.8) | 1976 (19.5) | 2505 (19.9) |  |
| >US$20,247 - 38,592 | 3516 (17.9) | 1973 (22.3) | 1543 (14.3) | <0.0001* |
| >US$38,592 - 72,292 | 3785 (22.3) | 1974 (26.3) | 1811 (18.9) |  |
| >US$72,292 | 5364 (40.1) | 1974 (31.8) | 3390 (46.9) |  |
| Total wealth, N (weighted %) | | | |  |
| ≤US$50,000 | 5834 (26.3) | 1992 (20.1) | 3842 (31.4) |  |
| >US$50,000 - 210,000 | 4192 (22.4) | 1968 (22.8) | 2224 (22.2) | <0.0001* |
| >US$210,00 - 649,000 | 3798 (24.7) | 1964 (25.8) | 1834 (23.9) |  |
| >US$649,000 | 3322 (26.6) | 1973 (31.4) | 1349 (22.6) |  |
| ***Nursing Home Expectations*** |  |  |  |  |
| Percent chance of moving into nursing home in the next 5 years (mean ± SE) | 15.2 ± 0.3 | 15.3 ± 0.3 | 12.2 ± 3.2 | 0.01* |
| Categories of expectations, N (weighted %) | | | |  |
| 0% | 3305 (38.9) | 3277 (38.9) | 28 (44.3) |  |
| 1 - 10% | 2005 (28.8) | 1990 (28.8) | 15 (31.3) |  |
| 11 - 40% | 1322 (17.8) | 1317 (17.9) | 5 (10.4) |  |
| 41 - 60% | 951 (10.8) | 947 (10.8) | 4 (14.0) |  |
| 61 - 100% | 366 (3.7) | 366 (3.7) | 0 (0.0) |  |
| ***Health Characteristics*** |  |  |  |  |
| Ever smoke, N (weighted %) | 9064 (53.5) | 4273 (53.8) | 4791 (53.3) | 0.18 |
| Ever drink alcohol, N (weighted %) | 9628 (61.6) | 4144 (56.6) | 5484 (65.7) | <0.0001* |
| At least one chronic condition (hypertension, diabetes, cancer, chronic lung disease, heart attack, stroke, arthritis), N (weighted %) | 7869 (48.2) | 3788 (50.9) | 4081 (46.0) | <0.0001* |
| At least one ADL limitation (among dressing, bathing, eating, toileting, walking/transferring), N (weighted %) | 3308 (15.6) | 1536 (16.9) | 1772 (14.6) | 0.63 |
| ***Mental Health Outcomes*** |  |  |  |  |
| Elevated depressive symptoms*,*  N (weighted %) | 1614 (9.3) | 586 (7.5) | 1028 (10.9) | <0.0001* |
| Major depressive episode*,*  N (weighted %) | 1305 (7.2) | 460 (5.8) | 845 (8.4) | <0.0001* |
| Passive suicidal ideation*,*  N (weighted %) | 1024 (5.7) | 396 (4.9) | 628 (6.3) | <0.0001* |

ADL=activity of daily living

^a^ Student’s t-tests were used for continuous variables, chi-squared tests were used for categorical variables

*p<0.05

**Supplementary Figure 1**. Flowchart of Main Sample and Leave-Behind Subsample Selection


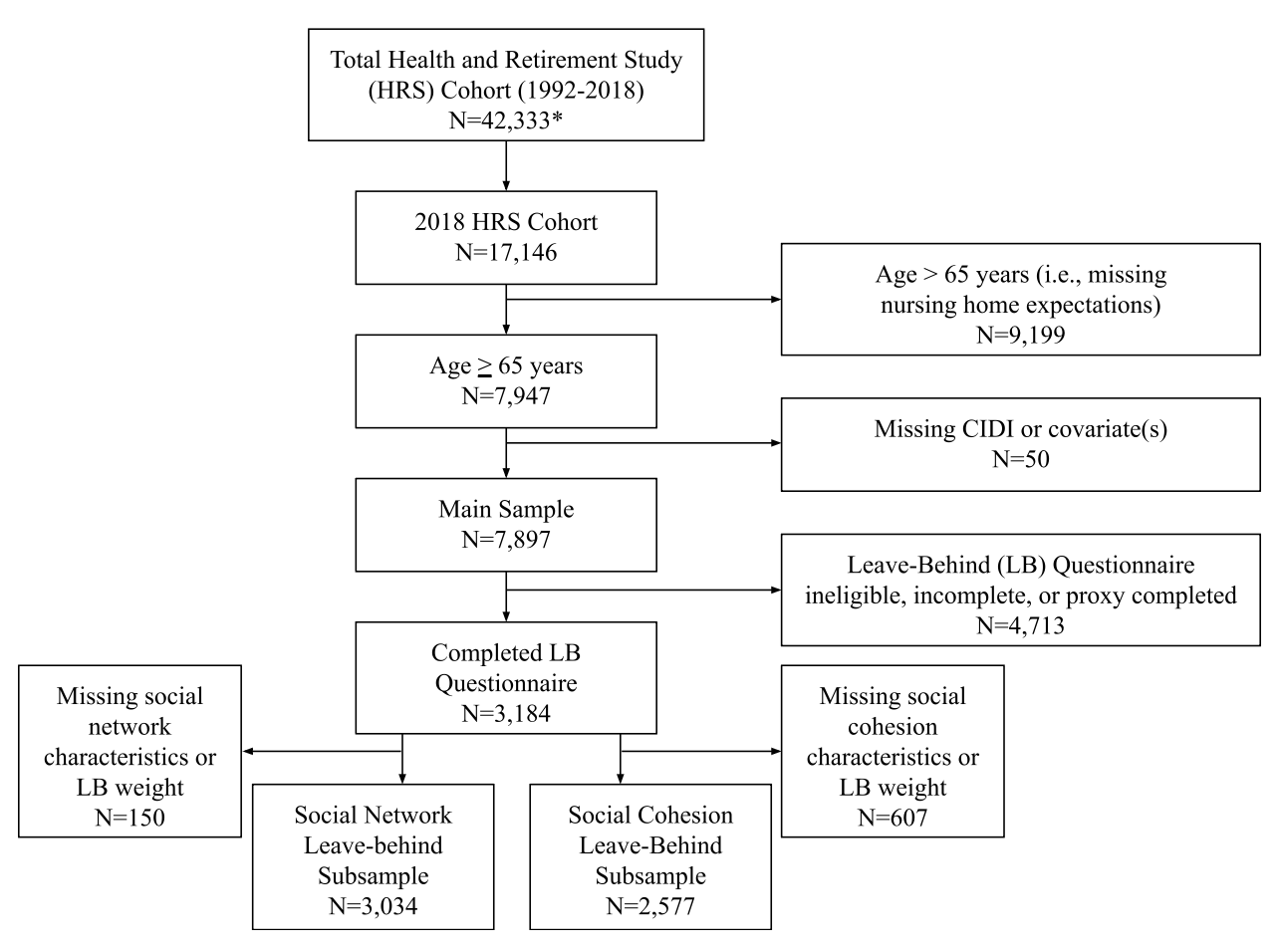


*Includes decedent respondents

CIDI=Composite International Diagnostic Interview-Short Form

**Supplementary Figure 2**. Distribution of Nursing Home Expectations Among Main Sample (N=7,897) (Health and Retirement Study, 2018)


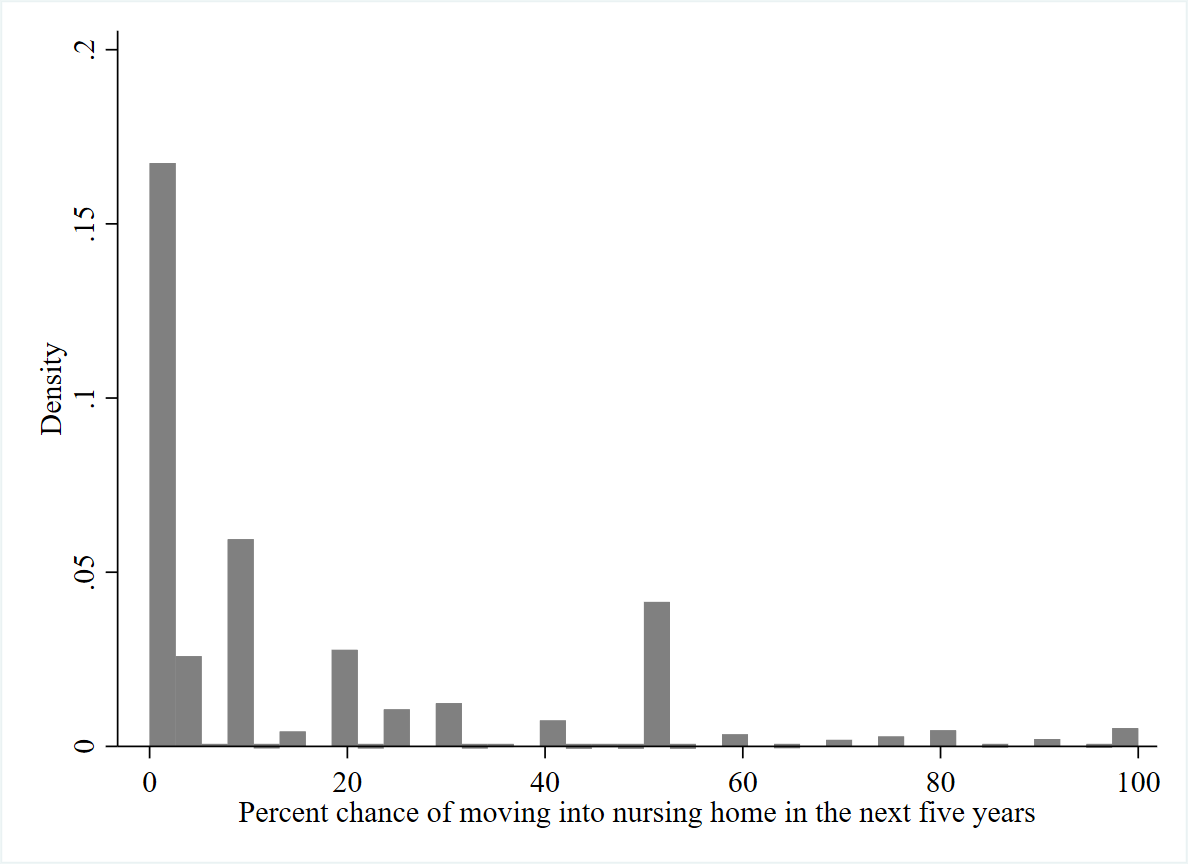

Supplement: Supplementary file 1 — Supplementary Material 1 [file 127_2025_2975_MOESM1_ESM.docx]
